# The Effect of Ex Vivo Human Serum from Liver Disease Patients on Cellular Protein Synthesis and Growth

**DOI:** 10.3390/cells11071098

**Published:** 2022-03-24

**Authors:** Sophie L. Allen, Alex P. Seabright, Jonathan I. Quinlan, Amritpal Dhaliwal, Felicity R. Williams, Nicholas H. F. Fine, David J. Hodson, Matthew J. Armstrong, Ahmed M. Elsharkaway, Carolyn A. Greig, Yu-Chiang Lai, Janet M. Lord, Gareth G. Lavery, Leigh Breen

**Affiliations:** 1 School of Sport, Exercise and Rehabilitation Sciences, University of Birmingham, Birmingham B15 2TT, UK; s.l.allen@bham.ac.uk (S.L.A.); a.seabright@bham.ac.uk (A.P.S.); j.quinlan@bham.ac.uk (J.I.Q.); c.a.greig@bham.ac.uk (C.A.G.); y.lai.1@bham.ac.uk (Y.-C.L.); 2 National Institute for Health Research, Birmingham Biomedical Research Centre, University Hospitals Birmingham NHS Foundation Trust, Birmingham B15 2TT, UK; a.dhaliwal.1@bham.ac.uk (A.D.); f.williams@bham.ac.uk (F.R.W.); matthew.armstrong@uhb.nhs.uk (M.J.A.); ahmed.elsharkaway@uhb.nhs.uk (A.M.E.); j.m.lord@bham.ac.uk (J.M.L.); gareth.lavery@ntu.ac.uk (G.G.L.); 3 Institute of Inflammation and Ageing, University of Birmingham, Birmingham B15 2TT, UK; 4 Liver Unit, Queen Elizabeth Hospital Birmingham, Nuffield House, Mindelsohn Way, Birmingham B15 2TH, UK; 5 Institute of Metabolism and Systems Research, University of Birmingham, Birmingham B15 2TT, UK; n.h.f.fine@bham.ac.uk (N.H.F.F.); d.j.hodson@bham.ac.uk (D.J.H.); 6 Centre for Endocrinology, Diabetes and Metabolism, Birmingham Health Partners, Birmingham B15 2TT, UK; 7 Oxford Centre for Diabetes, Endocrinology and Metabolism (OCDEM), NIHR Oxford Biomedical Research Centre, Churchill Hosptial, Radcliffe Department of Medicine, University of Oxford, Oxford OX3 7LE, UK; 8 MRC-Versus Arthritis Centre for Musculoskeletal Ageing Research, University of Birmingham, Birmingham B15 2TT, UK; 9 Department of Biosciences, Nottingham Trent University, Nottingham NG1 8NS, UK

**Keywords:** chronic liver disease, sarcopenia, leucine, mitochondria, protein breakdown

## Abstract

Sarcopenia is a common complication affecting liver disease patients, yet the underlying mechanisms remain unclear. We aimed to elucidate the cellular mechanisms that drive sarcopenia progression using an in vitro model of liver disease. C2C12 myotubes were serum and amino acid starved for 1-h and subsequently conditioned with fasted ex vivo serum from four non-cirrhotic non-alcoholic fatty liver disease patients (NAFLD), four decompensated end-stage liver disease patients (ESLD) and four age-matched healthy controls (CON) for 4- or 24-h. After 4-h C2C12 myotubes were treated with an anabolic stimulus (5 mM leucine) for 30-min. Myotube diameter was reduced following treatment with serum from ESLD compared with CON (−45%) and NAFLD (−35%; *p* < 0.001 for both). A reduction in maximal mitochondrial respiration (24% and 29%, respectively), coupling efficiency (~12%) and mitophagy (~13%) was identified in myotubes conditioned with NAFLD and ESLD serum compared with CON (*p* < 0.05 for both). Myostatin (43%, *p* = 0.04) and MuRF-1 (41%, *p* = 0.03) protein content was elevated in myotubes treated with ESLD serum compared with CON. Here we highlight a novel, experimental platform to further probe changes in circulating markers associated with liver disease that may drive sarcopenia and develop targeted therapeutic interventions.

## 1. Introduction

A loss of muscle mass, quality and strength, termed sarcopenia, is a common complication affecting ~25–70% of cirrhotic patients [1] and is considered a major component of malnutrition [2,3]. Sarcopenic cirrhotic patients have been shown to have an increase in pre- and post-liver transplant complications including infections, length of hospital stay [4], mortality [5] and a decrease in quality of life (QoL) [6]. Despite the recognized clinical complications and socio-economic burden of sarcopenia in chronic liver disease, the underlying mechanisms remain poorly understood [7].

Muscle atrophy is underscored by a negative net muscle protein balance arising from differences in muscle protein synthesis (MPS) and muscle protein breakdown (MPB). Impaired muscle protein turnover in chronic liver disease patients may be driven by hyperammonemia [8], inflammation [9], alcohol [10], physical inactivity [11] and insulin resistance [12]. Limited evidence in humans suggests that end stage liver disease (ESLD) patients experience a diminished MPS and anabolic signaling response to amino acid provision [13] compared with healthy individuals. Whole-body and muscle protein breakdown are also reportedly elevated in cirrhotic patients compared with healthy individuals, alongside increased markers of autophagy [14,15]. Finally, the myogenesis inhibitor, myostatin, has been found to be elevated in cirrhosis [8] and may be linked to alterations in protein turnover [9]. Given the paucity of evidence from human studies, the mechanisms of sarcopenia in liver disease patients across different etiologies and disease stages remain unclear. This is compounded by safety concerns around serial muscle biopsy sampling in liver disease patients due to their altered coagulation status and thrombocytopenia [14].

Beyond in vivo human studies, alternative in vitro [8] and in vivo [16] models of ESLD-like muscle atrophy have been developed through the use of ammonia treatment. These models highlight reductions in myotube atrophy in vitro [8] and muscle mass in portacaval anastomosis (PCA) rats [16]. However, the physiological relevance of in vivo animal and in vitro cell models of liver disease has been questioned [17]. Recently, an in vitro model in which C2C12 cells were conditioned with ex vivo human serum was used to demonstrate differences in cell morphology and MPS in the context of age-related sarcopenia [18], with findings aligned to evidence from in vivo human studies [19]. This novel experimental approach offers a potential avenue to probe mechanisms of sarcopenia in liver disease patients across different etiologies (e.g., alcohol-related and non-alcoholic fatty liver disease; NAFLD) and stages of disease severity (cirrhotic and non-cirrhotic).

Alterations in muscle protein turnover in chronic liver disease may arise due to a number of contributing factors, including impairments in mitochondrial function [20]. Specifically, mitochondrial dysfunction may contribute to a reduction in MPS through the diminution of tricarboxylic acid cycle intermediates and ATP synthesis, as shown with in vitro and in vivo hyperammonemia treatment [20]. This reduction in ATP may impair MPS, as translation initiation is known to be an energy intensive process [21]. Additionally, hyperammonemia has been shown to increase reactive oxygen species (ROS) and oxidative damage in rats and cirrhotic patients [20]. This increase in ROS may in turn lead to tissue injury and subsequent muscle loss [20]. Furthermore, the removal of damaged mitochondria, termed mitophagy has been implicated in age-related sarcopenia [22] and cancer cachexia [23], but has not been investigated in the context of chronic liver disease.

The aim of the present study was to use a physiologically relevant in vitro model to elucidate the mechanisms of sarcopenia progression in chronic liver disease, through conditioning C2C12 skeletal muscle cells with ex vivo human serum from decompensated, alcoholic related ESLD and non-cirrhotic NAFLD patients. To achieve this, we investigated myotube diameter and the regulatory mechanisms of myotube morphology, including mitochondrial respiration and mitophagy in response to 24 h of treatment with patient serum, as well as markers of MPS and proteolysis in response to a 4 h treatment with patient serum, with and without a 30 min leucine treatment. We hypothesized that serum from ESLD patients would lead to the development of myotube atrophy, alongside impairments in mitochondrial function, MPS and elevated proteolysis in comparison to myotubes treated with serum from non-cirrhotic NAFLD and age-matched controls (CON).

## 2. Materials and Methods

### 2.1. Participant Characteristics and Ethical Approval

Four male decompensated ESLD, four male non-cirrhotic NAFLD and four male age-matched healthy control participants provided their written, informed consent to participate in this study. CON participants were screened with a general health questionnaire and deemed eligible to participate. CON participants were expected to be normotensive (<140/90), free from metabolic disease conditions (i.e., type II diabetes and hyperlipidemia) and chronic inflammatory disease (i.e., chronic liver disease, inflammatory bowel disease and rheumatoid arthritis). ESLD patients with cirrhosis, who were assessed for liver transplant and non-cirrhotic NAFLD patients, were recruited after screening in a clinical setting. Presence of cirrhosis in ESLD was confirmed through serological and radiological confirmation, with or without a liver biopsy. All ESLD patients had alcohol-related liver disease (ArLD) as the underlying etiology. The non-cirrhotic NAFLD patients were recruited through a dedicated liver clinic and a diagnosis of cirrhotic was excluded in these by liver biopsy and elastography of <12 kPa in line with previous findings [24]. A full list of inclusion and exclusion criteria can be found in Table 1. Patients were not assessed for sarcopenia prior to recruitment to this study in order to allow for the assessment of prevalence to be determined in the ESLD and NAFLD cohorts. While we appreciate that ESLD patients with an NAFLD etiology would have been more appropriate, these cohorts were selected due to a pause in research completion during the COVID-19 pandemic. Consequently, we did not have access to blood samples from NAFLD patients with cirrhosis. Ethical approval for this study was obtained through the local ethics committee at the University of Birmingham (ERN_19-0831) for the recruitment of CON participants and the Health Research Authority—West Midlands Solihull Research Ethics Committee Authority (REC reference: 18/WM/0167) for the recruitment of NAFLD and ESLD patients and conformed to the standards set by the Declaration of Helsinki. This was a registered clinical trial (clinicaltrials.gov (accessed on 23 February 2022), ID: NCT04734496).

### 2.2. Study Design

All participants reported to the laboratory after an overnight fast with a minimum of at least 4–6 h adhered to and were asked to refrain from the consumption of caffeine on the morning of the trial. In addition, for 24-h prior to their laboratory visit participants were also asked to refrain from strenuous exercise. Upon arrival, a fasted venous blood sample was obtained. Blood samples were collected in a serum separator vacutainer (BD Biosciences, Oxford, UK) which was allowed to stand at room temperature for 30 min to allow the blood to clot. Vacutainers were then centrifuged at 3000× *g* for 10 min at 4 °C to obtain serum. Serum was stored at −80 °C until required. Finally, participants underwent basic body composition (body mass index (BMI), fat free mass (FFM), whole body fat mass (WBFM), body fat (BF) percentage, skeletal muscle mass (SMM)) and functional strength assessment through handgrip strength (HGS), as previously described [18].

### 2.3. Experimental Procedures

#### Blood Analyses

Serum C-reactive protein (CRP) (DCRP00), interleukin-6 (IL-6) (D6050) and insulin (DINS00) concentrations were determined using commercially available enzyme-linked immunosorbent assay (ELISA) kits following manufacturer’s instructions (R&D systems Inc., Minneapolis, MN, USA). Serum glucose concentrations were measured following manufacturer’s instructions using a Glucose-Glo™ Assay (J6021, Promega Corporation, Madison, WI, USA). Serum ammonia concentrations were measured utilizing an ammonia assay kit (ab83360; abcam, Cambridge, UK).

### 2.4. Cell Culture

Mouse skeletal muscle C2C12 cells (passage numbers 10–12) were obtained from the American Type Culture Collection (ATCC, Manassas, VA, USA). Cells were cultured, seeded and differentiated in Dulbecco’s Modified Eagle Medium (DMEM) (11966025, Gibco, Thermo Fisher Scientific, Leicestershire, UK), supplemented with, 5 mM glucose (G7021, Sigma-Aldrich, Poole, UK), 1 mM GlutaMAX^TM^ (35050-038, Gibco), 1 mM sodium pyruvate (11360070, Gibco) and 1% (*v/v*) penicillin/streptomycin (P/S) (15070-063, Gibco) with either 10% (*v/v*) fetal bovine serum (FBS) (F9665, Sigma-Aldrich) to allow for cell proliferation and 2% (*v/v*) horse serum (16050, Gibco) for cell differentiation as described previously [18]. C2C12s were maintained in standard conditions (5% CO_2_, 100% humidity and 37 °C) throughout experiments. For myotube diameter and respirometry analysis, on day 6 of differentiation, myotubes were incubated with DMEM as outlined above with 10% ex vivo human serum from CON, NAFLD and ESLD patients for 24 h. In order to investigate MPS, anabolic and catabolic signaling, myotubes were nutrient and serum starved in amino acid and serum free media (D9800-13, US Biological, Salem, MA, USA) for 1 h, following which they were treated with media containing 10% ex vivo human serum from CON, NAFLD, or ESLD subjects for 4 h. This preconditioning time period and serum dilution was selected based upon previous work within our laboratory as described in detail previously [18]. Myotubes were treated with serum from 4 participants to provide 4 biological replicates. This sample size was selected based upon previous work from our laboratory [18] and others [25]. All experiments were repeated in triplicate with *n* = 4 per group to provide 3 technical replicates per treatment.

### 2.5. Myotube Diameter

Following a 24 h incubation, media were removed and myotubes were fixed in 2% paraformaldehyde, followed by incubation with the primary antibody Desmin (1:100, D8281, Sigma-Aldrich) and the secondary antibody (1:200, Alexa Fluor Plus 488 goat anti-rabbit IgG H+L, A32731, Thermo Fisher Scientific) as previously described [18]. Nuclei were stained with DAPI (1:5000, 4083, Cell Signaling Technology (CST), Leiden, The Netherlands). Image acquisition was performed using a fluorescent microscope (Leica DMI6000B, Leica Microsystems, Wetzlar, Hessen, Germany) after an overnight incubation. For analysis, at least 100 myotubes were included from approximately 10 fields of view [18]. To calculate myotube diameter, the average of five measurements taken at equal increments along each myotube were used. Nuclear fusion index (NFI) was calculated based upon the number of nuclei contained in myotubes with three or more nuclei, expressed relative to the total number of nuclei in a field of view [26]. ImageJ software V 1.53k (US National Institutes of Health, Bethesda, MD, USA) was used for analysis.

### 2.6. Muscle Protein Synthesis

MPS was assessed using the surface sensing of translation (SUnSET) technique [27]. Briefly, the SUnSET technique utilizes immunoblotting to assess the incorporation level of the antibiotic puromycin, a tyrosyl-tRNA analogue (P8833, Sigma-Aldrich) into new synthesized peptide chains [27]. Puromycin (1 μM) was added to the media and incubated for the final 30 min of treatment. In a subset of samples, 5 mM leucine was added for 30 min at the end of the 4 h conditioning period to assess the anabolic response to leucine as previously described [18]. Subsequently, myotubes were washed with ice-cold phosphate-buffered saline (PBS) (Gibco, 10010015) 3-times over ice. RIPA lysis buffer (Merck Millipore, Watford, UK) (150 μL) was added to each well. After 20 min, cellular protein lysates were collected and centrifuged at 18,000× *g* for 15 min at 4 °C.

### 2.7. Immunoblotting

The concentration of protein in lysates was determined using a DC protein assay (Bio-Rad, Hercules, CA, USA) using a FLUOstar Omega plate reader (BMG Labtech, Aylesbury, UK) and equal amounts of protein (15–30 μg) from each sample were added to 1× Laemmli SDS sample buffer. Protein was then separated on 4–20% linear graded pre-cast gels (5671095, Bio-Rad) by SDS-PAGE and then transferred at 100 V for 1 h to a nitrocellulose membrane (Whatman, Dassel, Germany). As a loading control, membranes were stained with Ponceau S solution and blocked with 5% milk or bovine serum albumin (BSA) diluted in Tris-buffered saline and 0.1% Tween-20 (TBST) for 1 h. Overnight, the membranes were incubated at 4 °C with the following primary antibodies: phospho-mTOR Ser2448 (#2971, CST), total mTOR (#2972, CST), phospho-eukaryotic elongation factor 2 (eEF2) Thr56 (#2331, CST), total eEF2 (#2332, CST), phospho-ribosomal protein S6 kinase beta-1 (p70S6K1) Thr389 (#9205, CST), total p70S6K1 (#9202, CST), phospho-protein kinase B (Akt) Ser473 (#3787, CST), total Akt (#9272, CST), phospho-RPS-6 Ser240/244 (#5364, CST), total RPS-6 (#2217, CST), phospho-4EBP-1 Thr37/46 (#9459, CST), total 4EBP1 (#9452, CST), mouse IgG2a monoclonal anti-puromycin (clone 12D10, 1:5000, Merck Millipore), total OXPHOS rodent cocktail (ab110413, abcam), myostatin (ab201954, abcam), muscle RING finger 1 (MuRF-1) (sc-398608, Santa Cruz Biotechnology, Dallas, TX, USA) and muscle atrophy F-box (MAFbx) (AM3141, ECM Biosciences, Versailles, KY, USA), LC3A/B (#12741, CST) and Caspase-3 (D3R6Y) (#14220, CST). The following day, membranes were washed for 15 min with TBST and incubated for 1 h with the following secondary antibodies: anti-rabbit IgG horseradish peroxidase (HRP)-conjugated secondary antibody (#7074, CST) with the exception of MAFbx (anti-rat IgG, HRP-linked antibody, #7077, CST), puromycin, OXPHOS and MuRF-1 (anti-mouse IgG, HRP-linked antibody, #7076, CST). Membranes were washed for 15 min in TBST prior to the quantification of protein content using Immobilon Western chemiluminescent HRP substrate (Merck Millipore). Images were captured on a G:BOX Chemi XT4 imager using GeneSys capture software (Syngene, Cambridge, UK), and band quantification was conducted using ImageJ software. The whole lane was quantified for puromycin protein content in order to account for the full range of proteins which incorporate puromycin [27]. Normalization of relative arbitrary units was conducted using the protein band stained with Ponceau S solution.

### 2.8. Mitochondrial Respirometry

Mitochondrial respiration was measured in intact, attached cells as described previously [28]. Briefly, C2C12s were seeded at 4.0 × 10^4^ cells·mL^−1^ on XFe24 cell culture plates (Seahorse Bioscience, Agilent Technologies, Manchester, UK) and were differentiated. After treatment, myotubes were washed in Agilent Seahorse XF Base DMEM supplemented with 5mM of glucose, 1 mM of sodium pyruvate, 2 mM of L-glutamine and 5 mM 4-(2-hydroxyethyl)-1-piperazineethanesulfonic acid (HEPES) (15630106, Thermo Fisher). Subsequently, cells were incubated for 50 min at 37 °C before transferring to a Seahorse XFe24 extracellular flux analyzer (controlled at 37 °C) for a 10-min calibration and 4 measurement cycles to allow for the recording of basal cellular respiration. Subsequently, oligomycin (1 μM), BAM 15 (3 μM), and a mixture of rotenone (1 μM) and antimycin A (2 μM) were added to sequentially inhibit ATP synthase, uncouple oxidative phosphorylation and determine non-mitochondrial rates of respiration. A total of 4 measurement cycles were recorded after the addition of each compound. For all respirometry experiments each measurement cycle involved a 1-min mix, 2-min wait, and 3-min measurement period. All data were normalized to the total protein content quantified by a DC protein assay [29].

### 2.9. Mitophagy Assay

C2C12 myoblasts stably expressing mCherry-GFP-mt-FIS1 were generated as previously described [29]. Myoblasts were seeded at a density of 6 × 10^4^ cells·mL^−1^ onto imaging dishes with a polymer coverslip bottom (Ibidi, Gräfelfing, Germany) and treated with serum from either CON, NAFLD or ESLD participants for 24 h. After treatment, cells were washed twice with PBS and fixed for 10 min in 3.7% formaldehyde with 200 mM HEPES (pH 7.0). Following fixation, cells were washed and incubated for a further 10 min in DMEM supplemented with HEPES (10 mM, pH 7.0) and washed with PBS. Subsequently, a mounting solution was added to the wells. After an overnight incubation in the dark, images were taken using a Crest X-Light spinning disk system coupled to a Nikon Ti-E base, 60×/1.4 NA (CFI Plan Apo Lambda) air objective and Photometrics Delta Evolve EM-CCD. Excitation was required during imaging and delivered at λ = 458–482 nm and λ = 563–587 for GFP and mCherry, respectively. Signals were detected at λ = 500–550 nm and λ = 602–662 respectively.

Mitophagy was quantified in single cells using the mean fluorescence intensity of mCherry/GFP measured in 25 cells across at least 15 fields of view for each condition using ImageJ software [29]. An increase in mCherry/GFP ratio is indicative of mitophagy at a whole cellular level. The mCherry/GFP ratio was normalized to CON treated samples.

### 2.10. Statistical Analysis

Statistical analysis was performed using GraphPad Prism V8 4.3. Data were tested for homogeneity of variances (Levene’s test) and normality (Shapiro–Wilk test). A one-way ANOVA was used to investigate differences in anthropometrics, body composition (FFM, SMM, FM, body fat %), HGS and plasma analytes/hormones (glucose, insulin, IL-6, CRP and serum ammonia) and the analysis of myotube diameter, NFI, respirometry, mitophagy and the protein content of OXPHOS. A 3 (group) × 2 (leucine vs. serum only) ANOVA was used to investigate differences in the protein content of anabolic and catabolic signaling and puromycin incorporation. Where the results of the one-way or two-way ANOVAs revealed a significant interaction or main effect, post hoc analysis t-tests were completed using Tukey’s HSD. Effect size was calculated using Cohen’s d for t-test and post hoc comparisons, while partial eta squared (η^2^) was used for omnibus tests. Statistical significance was set at *p* < 0.05. Data are reported as mean ± standard deviation in tables and as the mean (cross), median (central horizontal line), 25th and 75th percentiles (box) and the minimum and maximum values (vertical lines) in figures.

## 3. Results

### 3.1. Body Composition and Strength 

Measures of anthropometrics, body composition and strength are shown in Table 2. No significant between-group difference was observed in age, body mass and BMI. No significant between-group difference was identified between FFM, WBFM, and skeletal muscle mass. However, BF percentage was significantly elevated in both NAFLD (100%, *p* < 0.01, d = 3.9) and ESLD patients (75%, *p* < 0.05, d = 2.6) compared with CON. No significant difference in HGS was identified between groups.

### 3.2. Blood Analysis

Serum ammonia concentration was significantly greater in ESLD patients compared with CON participants (*p* = 0.01, d = 3.5). No significant difference was found between CON and NAFLD patients, and NAFLD and ESLD patients (Table 2). Serum IL-6 was significantly greater in ESLD patients compared with CON (*p* = 0.02, d = 2.7). Serum CRP was significantly increased in ESLD patients compared with CON (*p* < 0.001, d = 7.0) and NAFLD (*p* < 0.001, d = 6.6) patients. No significant difference in CRP concentration was found between CON and NAFLD patients. Serum insulin was significantly greater in ESLD patients in comparison to CON participants (158% *p* = 0.02, d = 2.2). No significant difference in serum insulin concentration was identified in NAFLD patients when compared to both CON and ESLD patients; however, a large effect size was observed for both (*p* = 0.3, d = 2.2; *p* = 0.2, d = 1.2 respectively). Serum glucose was not significantly different between groups. HOMA-IR was significantly increased within ESLD patients in comparison to CON participants (159%, *p* = 0.02, d = 2.6). No significant difference in serum IL-6, and HOMA-IR was identified in NAFLD compared with both ESLD and CON participants.

### 3.3. Myotube Diameter and Nuclear Fusion Index

**Figure 1:**
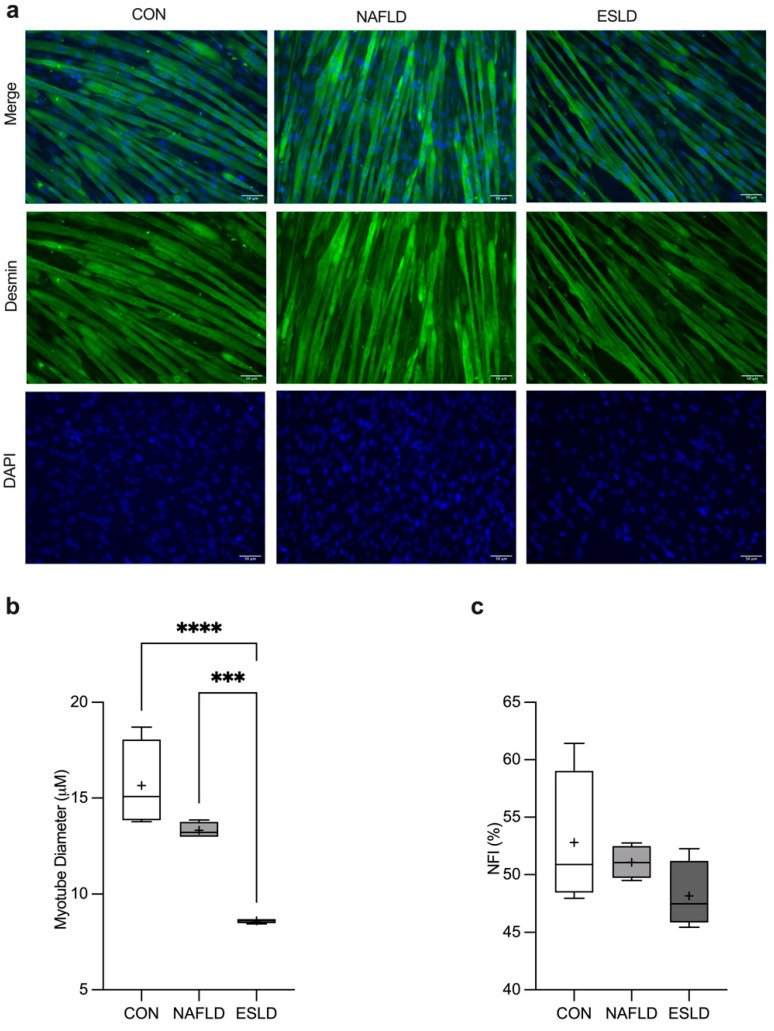
Serum from NAFLD and ESLD patients induces myotube atrophy. (**a**) Representative images illustrating atrophy in myotubes treated with ex vivo serum from non-alcoholic fatty liver disease (NAFLD) and end stage liver disease (ESLD) patients, in comparison to myotubes treated with serum from age-matched control participants (CON), (**b**) mean myotube diameter, (**c**) mean nuclear fusion index (NFI). Data are expressed as the mean (cross), median (central horizontal line), 25th and 75th percentiles (box) and the minimum and maximum values (vertical lines), with *n* = 4 per group and each data point corresponding to the average of 3 technical repeats. **** *p* < 0.0001, *** *p* < 0.001.

A one-way ANOVA revealed a significant main effect between groups (*p* < 0.001, η^2^ = 0.86, F = 28.81) (Figure 1b, Supplementary Figure S1). Myotube diameter was significantly reduced in response to conditioning with serum from ESLD patients compared with CON (45%, *p* < 0.0001, d = 5.9) and NAFLD (35%, *p* < 0.002, d = 17.4) patients. Myotube diameter was numerically lower in myotubes conditioned with serum from NAFLD compared with CON patients, but did not reach significance (15%, *p* = 0.08, d = 1.7). No significant difference in NFI was identified between experimental groups (Figure 1c, Supplementary Figure S1).

### 3.4. Muscle Protein Synthesis and Anabolic Signaling

**Figure 2:**
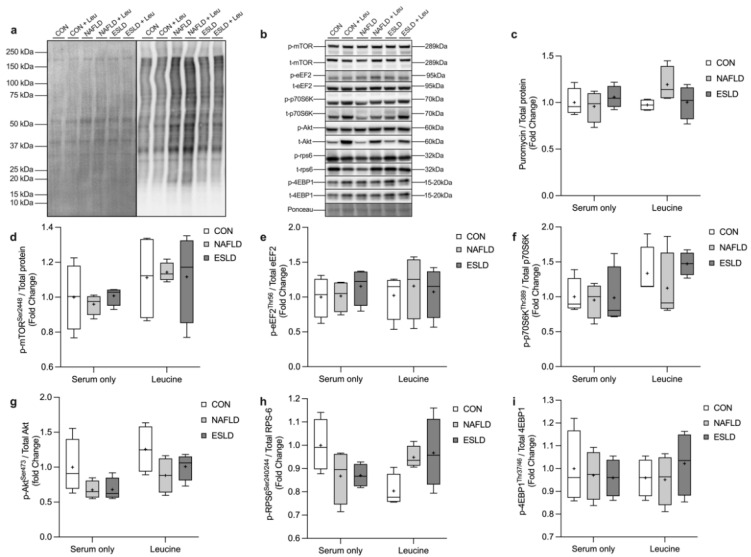
Measures of MPS and anabolic signaling in response to treatment with CON, NAFLD and ESLD serum. Myotubes were treated for 4 h with ex vivo serum from age-matched control (CON), non-alcoholic fatty liver disease (NAFLD) and end stage liver disease (ESLD) patients. (**a**) Representative western blot for puromycin and total protein, (**b**) representative western blots for anabolic signaling targets and loading control, (**c**) puromycin incorporation, (**d**) phospho-mTOR (Ser2448)/total-mTOR, (**e**) phospho-eEF2 (Thr56)/total-eEF2, (**f**) phospho-p70S6K (Thr389)/total-p70S6K, (**g**) phospho-AktSer473/total-Akt, (**h**) phospho-RPS6 (Ser240/244)/total-RPS6, (**i**) phospho-4EBP-1 (Thr37/46)/total-4EBP-1. Data are expressed as the mean (cross), median (central horizontal line), 25th and 75th percentiles (box) and the minimum and maximum values (vertical lines), with *n* = 4 per group and each data point corresponding to the average of 3 technical repeats.

After conditioning myotubes with serum from CON, NAFLD and ESLD patients, we found no significant difference in MPS between- or within-groups in basal conditions or following the addition of leucine (Figure 2). Additionally, we found no significant difference in the phosphorylation of mTOR, 4EBP-1 and eEF2 between groups, with and without leucine treatment (Figure 2). A two-way ANOVA revealed a significant interaction effect between the effects of group and treatment in the phosphorylation of RPS6 (F (2, 18) = 5.6, *p* = 0.01). Additionally, a significant main effect for treatment (i.e., serum only vs. leucine) was identified for the phosphorylation of p70S6K (F (1, 18) = 5.4, *p* = 0.03). Furthermore, a significant main effect for treatment (F (1, 18) = 6.1, *p* = 0.02) and group (i.e., CON, NAFLD, ESLD) (F (2, 18) = 4.1, *p* = 0.04) was identified for the phosphorylation of Akt. Despite the interaction and main effects identified here, post hoc analysis revealed no significant difference in the phosphorylation of RPS6, p70S6K and Akt between groups with and without leucine (Figure 2).

### 3.5. Proteolytic Signaling

**Figure 3:**
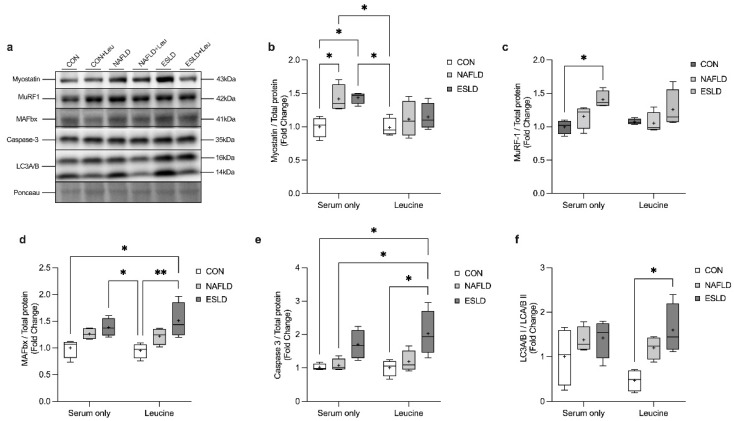
Markers of catabolic signaling are elevated within myotubes treated with serum from ESLD patients for 4 h and a 30 min treatment with leucine. Myotubes were treated with ex vivo human serum from age-matched control (CON), non-alcoholic fatty liver disease (NAFLD) and end stage liver disease (ESLD) patients for 4-h, with and without a 30-min treatment with 5 mM leucine. (**a**) Representative western blot images of catabolic signaling markers, (**b**) myostatin, (**c**) MAFbx, (**d**) MuRF-1, (**e**) caspase-3, (**f**) LC3A/B. Data are expressed as fold change in comparison to the CON. Data are expressed as the mean (cross), median (central horizontal line), 25th and 75th percentiles (box) and the minimum and maximum values (vertical lines), with *n* = 4 per group and each data point corresponding to the average of 3 technical repeats. * *p* < 0.05, ** *p* < 0.01.

A two-way ANOVA revealed a significant main effect for group for the protein content of myostatin (F (2, 18) = 6.3, *p* = 0.008), MuRF-1 (F (2, 18) = 6.9, *p* = 0.006), MAFbx (F (2, 18) = 12.1, *p* = 0.0005), Caspase-3 (F (2, 18) = 6.7, *p* = 0.007) and LC3A/B (F (2, 18) = 11.8, *p* = 0.0005) (Figure 3). Additionally, a significant main effect for treatment was identified for the protein content of myostatin (F (1, 18) = 7.1, *p* = 0.02).

Post-hoc analysis revealed that myostatin protein content was significantly increased in myotubes treated with serum from NAFLD (42%, *p* = 0.05, d = 2.5) and ESLD (44%, *p* = 0.04, d = 3.6) compared with those treated with serum from CON. Similarly, MuRF-1 protein content was significantly increased in myotubes treated with serum from ESLD patients compared with CON (*p* = 0.03, d = 3.5). No difference in the protein content of MAFbx, Caspase 3 and LC3A/B was identified in myotubes in basal conditions between groups. In response to the addition of leucine we found a further increase in MAFbx (51%) and Caspase 3 (99%) above basal conditions in ESLD treated myotubes, which was significantly greater than myotubes treated with CON serum, in a basal non-leucine state (*p* = 0.02, d = 2.0; *p* = 0.02, d = 2.6 respectively). Similarly, in the presence of leucine stimulation myotubes treated with serum from ESLD patients showed elevated levels of MAFbx (59%), Caspase 3 (102%) and LC3A/B (237%) compared with CON treated myotubes with leucine (*p* = 0.007, d = 2.4; *p* = 0.01, d = 2.2; *p* = 0.02, d = 2.8, respectively). The protein content of MAFbx was also found to be elevated in myotubes treated with serum from ESLD patients compared with those treated with serum from CON with leucine (46%, *p* = 0.05, d = 2.8). Additionally, in comparison to CON leucine-stimulated myotubes, NAFLD and ESLD basal conditions showed an increase in myostatin protein content (12%, *p* = 0.04, d = 0.6, 23%, *p* = 0.03, d = 1.4, respectively). The protein content of caspase 3 was also shown to be elevated in myotubes treated with ESLD serum and leucine compared with NAFLD serum alone (173%, *p* = 0.03, d = 2.2). No difference in the protein content of myostatin, MuRF-1, MAFbx and LC3A/B was detected between myotubes treated with serum from NAFLD and ESLD patients without the presence of leucine.

### 3.6. Mitochondrial Respiration

**Figure 4:**
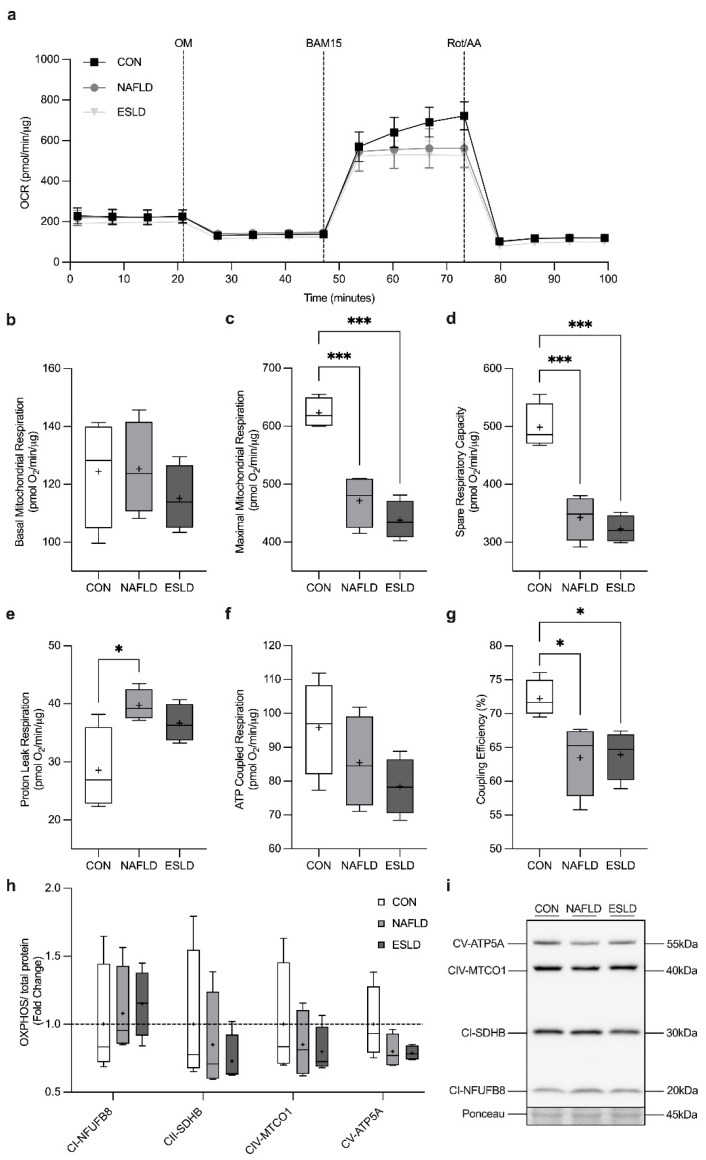
Serum from NAFLD and ESLD patients induces impairments in mitochondrial respiration, despite no changes in markers of mitochondrial protein content. (**a**) Raw trace of oxygen consumption rate (OCR) for myotubes treated with ex vivo serum from non-alcoholic fatty liver disease (NAFLD), end stage liver disease (ESLD) and age-matched controls (CON), (**b**) basal mitochondrial respiration was assessed prior to the addition of oligomycin (OM), an ATP synthase inhibitor and represents the energetic demand of the cell in baseline conditions, (**c**) maximal mitochondrial respiration was calculated after the addition of the uncoupler BAM-15, (**d**) spare respiratory capacity indicates the ability of a cell to respond to an increase in energetic demand (**e**) proton leak respiration represents the portion of basal respiration not associated with ATP production (**f**) ATP coupled respiration was calculated upon the addition of OM, (**g**) coupling efficiency was calculated as the percentage of respiration accounted for by ATP production, (**h**) quantification of OXPHOS/total protein, (**i**) representative western blot of OXPHOS. Respirometry data were normalized to protein content. Western blot data are expressed as fold change in comparison to CON. Data are expressed as the mean (cross), median (central horizontal line), 25th and 75th percentiles (box) and the minimum and maximum values (vertical lines), with *n* = 4 per group and each data point corresponding to the average of 3 technical repeats. *** *p* < 0.001, * *p* < 0.05.

We assessed oxygen consumption through the completion of a mitochondrial stress test after a 24-h treatment with serum from CON, NAFLD and ESLD cohorts (Figure 4a). No significant difference was identified in basal mitochondrial respiration (Figure 4b). However, a significant main effect was identified in maximal mitochondrial respiration (*p* < 0.001, η^2^ = 0.86, F = 30.07) (Figure 4c). Indeed, maximal mitochondrial respiration significantly decreased in myotubes treated with serum from NAFLD (24%, *p* < 0.001, d = 4.20) and ESLD patients (30%, *p* < 0.001, d = 6.25) compared with CON, with no significant difference between NAFLD and ESLD treated myotubes. Furthermore, a significant main effect of spare respiratory capacity was identified between groups (*p* < 0.001, η^2^ = 0.87, F = 31.21) (Figure 4d). Spare respiratory capacity was significantly greater in myotubes treated with serum from CON, in comparison to NAFLD (31%, *p* < 0.001, d = 4.02) and ESLD (35%, *p* < 0.001, d = 5.61). No significant difference was identified between myotubes treated with NAFLD and ESLD serum. A significant main effect was identified for proton leak respiration between groups (*p* = 0.02, η^2^ = 0.57, F = 5.9) (Figure 4e). Proton leak respiration was significantly increased in myotubes treated with serum from NAFLD patients compared with CON (39%, *p* = 0.02, d = 2.29). While a large effect size was observed, no significant difference in proton leak respiration was identified between CON and ESLD treated myotubes (28%, *p* = 0.09, d = 1.73). No significant difference was identified between NAFLD and ESLD serum treated myotubes. Similarly, a significant main effect was identified between groups in coupling efficiency (*p* = 0.02, η^2^ = 0.56 F = 5.8) (Figure 4g). Coupling efficiency was significantly decreased in myotubes treated with serum from NAFLD (12%, *p* < 0.05, d = 2.15) and ESLD patients (12%, *p* < 0.05, d = 2.6) compared with CON, with no significant difference between myotubes treated with serum from NAFLD and ESLD patients. No significant difference in ATP coupled respiration was identified between groups (Figure 4f).

### 3.7. Mitophagy

**Figure 5:**
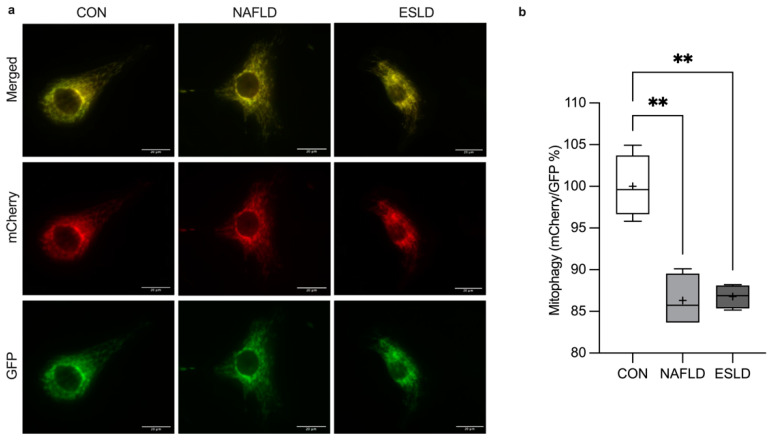
Serum from NAFLD and ESLD patients induces a reduction in mitophagy. (**a**) Representative images illustrating that treatment of mitoQC cells with ex vivo serum from patients with non-alcoholic fatty liver disease (NAFLD) and end stage liver disease (ESLD) in comparison to age-matched control (CON) patients in C2C12 myoblasts stably expressing mCherry-GFP-FIS1101-152, (**b**) quantification of mitophagy, expressed as mCherry/GFP. Increases in the relative mCherry/GFP ratio assessed by fluorescence intensity indicate mitophagy. Data are represented a fold change from CON. Data are expressed as the mean (cross), median (central horizontal line), 25th and 75th percentiles (box) and the minimum and maximum values (vertical lines), with each data point corresponding to the mean of 25 measurements per condition. An *n* = 4 was utilized within each group. ** *p* < 0.01.

Mitophagy was assessed using a mitophagy reporter cell line (mitoQC) in C2C12 skeletal muscle cells, in which C2C12s express a stable functional insert tandem mCherry-GFP tag, which is fused to the mitochondrial targeting sequence of FIS1 (residues 101–152) [30]. Under basal conditions, the mitochondria fluoresce both red (mCherry) and green (GFP), which when merged appear gold in color. However, mitophagy induces an acidic environment within the lysosomes which quench the signal of GFP, but not mCherry [29]. Consequently, an increase in mCherry signal, thus the mCherry/GFP ratio measured by the intensity of fluorescence, occurs within the mitochondrial network indicating mitophagy. A one-way ANOVA revealed a significant main effect between groups for the analysis of mCherry/GFP (*p* < 0.001, η^2^ = 0.86, F = 27.19) (Figure 5a). Specifically, in comparison to myoblasts treated with serum from CON there was a significant reduction in the level of mitophagy, as shown by a decrease in the ratio of mCherry/GFP reported in myoblasts treated with serum from NAFLD (14%, *p* < 0.001, d = 3.9) and ESLD (13%, *p* < 0.001, d = 5.1) patients. No significant difference in mitophagy was evident between myotubes treated with serum from NAFLD and ESLD patients.

### 3.8. Mitochondrial Content

Treatment with serum from CON, NAFLD and ESLD patients showed no significant difference in markers of mitochondrial protein content measured through the expression of oxidative phosphorylation (OXPHOS) subunits (I-V) (Figure 4h).

## 4. Discussion

The use of an in vitro model utilizing ex vivo human serum from CON, ESLD and NAFLD patients to condition C2C12 skeletal muscle cells, provides a model to investigate the potential mechanisms which underpin the progression of sarcopenia in chronic liver disease patients of different underlying etiologies and clinical stages of disease. This model is advantageous as it circumvents concerns associated with muscle biopsies in ESLD patients [14] and provides a more physiologically relevant basis to study mechanisms of muscle atrophy compared with typical in vitro approaches [18]. In line with our hypotheses, we demonstrated that conditioning myotubes with serum from ESLD patients induced myotube atrophy compared with those treated with CON serum. Mechanistically, we identified mitochondrial dysfunction in myotubes treated with serum from NAFLD and ESLD patients compared with CON. Additionally, we identified an increase in myostatin and proteolytic markers in myotubes treated with ESLD serum compared with CON. Fasted and fed state MPS and anabolic signaling did not differ between groups. Collectively, these data demonstrate potential mechanisms of sarcopenia progression in ESLD and NAFLD patients.

After 24 h of conditioning with ex vivo human serum, we found that serum from ESLD patients led to a significant decrease in myotube diameter in comparison to CON and NAFLD treated myotubes (~45% and 35%, respectively). This observation is consistent with in vivo animal work, highlighting muscle atrophy in a PCA rat model of cirrhosis [31]. Potential underlying factors that may contribute to the observed myotube atrophy and sarcopenia in ESLD include increased inflammation [32,33] and ammonia [15], which are exacerbated with liver disease progression and were markedly greater in ESLD compared with CON and NAFLD in the present study [9]. Further in vitro and in vivo research is required to understand the systemic factors which may contribute to myotube atrophy and sarcopenia progression in chronic liver disease, including other etiologies (e.g., alcohol-related liver disease; ArLD).

The observed myotube atrophy in response to ESLD patient serum would undoubtedly be underscored by dysregulation of muscle protein turnover. In contrast to our hypothesis, we did not observe an impairment in fasted-state MPS or markers of anabolic signaling in response to ESLD serum treatment compared with CON and NAFLD patients. These findings contrast with previous work showing a reduction in fasted-state anabolic signaling in cirrhotic ArLD patients compared with healthy controls [14]. The lack of fasted-state anabolic signaling impairment identified in this study could be partially explained by the differences between experimental models, patient status (i.e., compensated vs. decompensated), anthropometrics and age. Although previous in vitro and in vivo animal ArLD models have demonstrated an impairment in mTORC1 signaling with ethanol treatment [10], patient donors with an ArLD etiology in the present study had abstained from alcohol prior to recruitment, which may explain the absence of any impairment in fasted-state anabolic signaling herein.

Alongside no differences in fasted-state MPS, we found no increase in leucine-stimulated MPS in any of the groups. As CON participants in this study had a mean age of ~65 years, it is plausible that the absence of a leucine-stimulated MPS or anabolic signaling response could be due to older age, as demonstrated in our recent work using this in vitro model [18]. Interestingly, previous in vivo work reported a similar increase in anabolic signaling in ArLD cirrhotic and healthy controls in response to leucine-enriched branched-chain amino acid (BCAA) ingestion [14], although the large dose of leucine provided in this study (7.5 g) may have masked any potential muscle anabolic resistance [34]. Nonetheless, it is surprising that we did not detect an impairment in postprandial MPS stimulation in ESLD or NAFLD compared with CON, particularly as BMI and body fat mass was greater in the liver disease cohorts and obesity exacerbates age-related muscle anabolic resistance to protein provision [32]. Notwithstanding, the present findings suggest that myotube atrophy in response to ex vivo ESLD serum treatment is not due to an impairment in fasted-sate or leucine-stimulated MPS or anabolic signaling. However, in vivo human studies of fasted and postprandial MPS in liver disease patients across different etiologies, are required to fully elucidate whether MPS dysregulation contributes to sarcopenia in chronic liver disease.

Despite the absence of any difference in MPS, we report an increase in the myogenesis inhibitor, myostatin, in myotubes treated with serum from NAFLD and ESLD patients. This finding is consistent with the observation that myostatin expression is increased in cirrhotic patients alongside reduced skeletal muscle mass [8]. Similarly, ammonia and ethanol treatment have been shown to increase myostatin expression and reduce myotube diameter in C2C12 [35]. Mechanistically, increased myostatin has been linked to the suppression of MPS and anabolic signaling [36]; however, we did not detect alterations in MPS herein. Myostatin has also been linked to an increase in MPB, through an increase in ubiquitin proteasome markers [37]. In cirrhotic patients, elevated myostatin has also been reported alongside increased autophagy and whole-body protein breakdown (WbPB) compared with healthy controls [14,15]. Similarly, we identified an increase in autophagy and the ubiquitin proteasome markers in ESLD treated myotubes compared with CON. We also observed an increase in MuRF-1 protein content in response to ESLD serum treatment, which has not been detected in earlier human studies [14,15]. Beyond fasted-state proteolytic signaling, BCAA treatment in ArLD cirrhotics has been reported to attenuate autophagy [14]. In contrast, markers of proteolysis were not altered by leucine treatment in the present study across groups. This discrepancy may be due to differences in signaling responsiveness in leucine-treated C2C12 cells and human skeletal muscle with orally ingested high-dose BCAA/leucine. Interestingly, despite elevated myostatin expression in ESLD and NAFLD compared with CON serum treated cells, markers of proteolysis differed between ESLD and CON only. This finding may partly explain why differences in myotube atrophy in NAFLD vs. CON did not reach significance, whereas ESLD serum treated cells experienced significant atrophy compared with NAFLD and CON. We acknowledge that the stage of disease severity differed between NAFLD and ESLD donors in this study, and that serum treated cells from decompensated cirrhotic NAFLD patients may have undergone greater proteolysis and myotube atrophy compared with the noncirrhotic NAFLD patients included herein. Taken together, the present findings suggest that myotube atrophy with ex vivo ESLD serum treatment, may be partially underpinned by elevated myostatin and markers of proteolysis. Although these purported mechanisms of myotube atrophy in ESLD require further exploration, interventions targeting myostatin and proteolytic pathway regulators may provide a promising approach to offset sarcopenia progression in chronic liver disease.

In the present study, we report for the first time that mitochondrial respiration and coupling efficiency was impaired in C2C12s treated with serum from NAFLD and ESLD patients compared with CON. Previously, reduced mitochondrial oxidative capacity has been observed in conditions of muscle atrophy including age-related sarcopenia [38] and cancer cachexia [39], but has not been studied in chronic liver disease. In vitro work has shown impairments in cellular respiration in response to ammonia [20] and ethanol [10] treatment, and reversal in the reduction of ATP content with ammonia removal from culture media. Expanding on these observations by using a physiologically relevant in vitro model, our novel findings support the notion that mitochondrial dysfunction could play an important role in sarcopenia progression in chronic liver disease. Additionally, we observed a reduction in mitophagy in both NAFLD and ESLD treated C2C12 mitoQC cells compared with CON. This is in line with previous in vivo work highlighting a reduction in mitophagy in the context of age-related sarcopenia [40] and NAFLD models [41]. Although the mechanisms of mitophagy across the chronic liver disease spectrum remain unclear, this process is considered to be beneficial for the clearance of damaged mitochondria [42]. Indeed, the inhibition of mitophagy leads to the accumulation of dysfunctional mitochondria [22]. As a consequence, this may explain the reduction in mitochondrial respiration observed in the present study.

Changes in mitochondrial respiration and mitophagy occurred independently of changes in mitochondrial protein content, similar to observations from an in vitro ArLD model [10]. Despite a similar degree of mitochondrial dysfunction in NAFLD and ESLD treated myotubes compared with CON, significant myotube atrophy was only present in ESLD treated myotubes. Previous in vivo mouse models of cancer cachexia have shown that mitochondrial dysfunction develops prior to the loss of muscle mass [23]. Therefore, it is plausible that mitochondrial dysfunction may arise at an early stage of liver disease (e.g., non-cirrhotic NAFLD patients) prior to accelerated sarcopenia and myotube atrophy that is observed in ESLD [43]. Additionally, ESLD patients were found to be insulin resistant in comparison to CON participants, a factor which has also been linked to the development of mitochondrial dysfunction [44]. It is worth noting that the mitochondrial dysfunction identified here may be related to increased myostatin, which can influence glucose metabolism and the development of insulin resistance [45]. Further exploratory research is required to understand the causes of mitochondrial dysfunction and potential links to impairments in muscle protein turnover that underlie sarcopenia in chronic liver disease.

Although this in vitro work provides a valuable platform to investigate the mechanisms which underpin muscle atrophy in ESLD, there are limitations to this approach. Firstly, we did not treat human primary skeletal muscle cells with serum from NAFLD and ESLD patients or CON individuals, but instead used C2C12 murine cells, therefore this approach does not allow for the potential role of peripheral factors i.e., intrinsic muscular and neural properties. Despite potential cross-species issues, this approach is beneficial for use when primary muscle cultures are unavailable from patient cohorts. As a result, future research should aim to replicate this work using human primary muscle cells, from the patient cohorts where possible, to investigate the potential links between intrinsic muscle factors and the systemic factors driving this atrophy shown in this study. Secondly, as an extensive blood analysis was not completed, the systemic factors which drive the myotube atrophy and/or mechanistic dysregulation identified in ESLD and NAFLD serum treated cells remain unclear. Whilst hyperammonemia [15], inflammation [9] and alcohol [10] may drive sarcopenia progression in chronic liver disease, other systemic factors including insulin resistance [9], decreased testosterone [46] and IGF-1 [47] may influence sarcopenia progression. Finally, beyond the mechanisms explored in the present study, others have suggested that increased oxidative stress [20,48], impaired ribosomal biogenesis [49] and satellite cell function [31] may contribute to the dysregulated muscle protein turnover in cirrhotic animals. It is likely that all of these factors may underpin the development of sarcopenia in ESLD, with the degree varying between individuals due to disease etiology and possibly gender [1] and lifestyle factors (diet, physical activity; [50,51]).

## 5. Conclusions

In conclusion, our data suggest that sarcopenia progression in ESLD may be linked to mitochondrial dysfunction, increased myostatin and potentially proteolysis. In vivo human studies are now required in order to confirm these observations and to resolve the systemic factors that drive sarcopenia across complex multi-faceted chronic liver disease pathologies and disease stage.

## Figures and Tables

**Table 1:** Inclusion and Exclusion Criteria.

	Inclusion Criteria	Exclusion Criteria
ESLD	1. Adults aged ≥ 18 years old 2. Male 3. Capable of providing written informed consent 4. Cirrhosis (defined by Child Pugh Scores A–C) 5. Radiological and serological evidence of cirrhosis +/− a liver biopsy 6. Listed on the UK transplant list	1. Lack of capacity to provide informed consent. 2. Previously undergone liver transplant 3. Diagnosed with cancer 4. Receiving biliary intervention 5. Enrolled in an intervention trial for liver disease
NAFLD	1. Adults aged ≥ 18 years old 2. Male 3. Capable of providing written informed consent 4. Significant fibrosis (F2–F4 Kleiner fibrosis stages) confirmed with liver biopsy 5. Fibroscan of < 12 kPa (to exclude cirrhosis)	1. Lack of capacity to provide informed consent 2. Cirrhosis (defined by Child Pugh scores A–C) 3. Listed on the UK transplant list 4. Fibroscan > 15 kPa
CON	1. Adults aged ≥ 18 years old 2. Male 3. Body mass index of 18.5–34.99 4. Capable of providing written informed consent Free from inflammatory chronic disease (i.e., good general health) Non-smoker Non-diabetic	1. Lack of capacity to provide informed consent. 2. Diagnosed with diabetes 3. Diagnosed with any inflammatory disease condition Smoker

**Table 2:** Donor Characteristics.

	CON (*n* = 4)	NAFLD (*n* = 4)	ESLD (*n* = 4)
Anthropometrics			
Age (years)	64.7 ± 10.0	61.8 ± 7.6	60.5 ± 1.7
Body mass (kg)	74.8 ± 7.6	108.4 ± 31.3	108.1 ± 18.5
Height (cm)	179.0 ± 7.4	171.6 ± 3.6	174.3 ± 4.3
BMI (kg m^−2^)	23.3 ± 1.6	37.1 ± 11.33	35.7 ± 7.3
FFM (kg)	61.4 ± 6.1	67.9 ± 11.4	73.2 ± 6.7
WBFM (kg)	13.4 ± 2.2	40.6 ± 20.1	34.9 ± 15.5
BF (%)	17.8 ± 1.9	35.7 ± 7.3 *	31.3 ± 8.7 *
Strength			
HGS (kg)	54.0 ± 9.8	41.1 ± 7.1	40.2 ± 8.2
Blood Analyses			
Fasting Serum Insulin (μIU/mL)	9.6 ± 4.1	16.88 ± 3.5	24.6 ± 10.1 *
Fasting Serum Glucose (mmol/L)	4.4 ± 0.1	4.3 ± 0.1	4.3 ± 0.1
HOMA-IR	1.8 ± 0.7	3.2 ± 0.6	4.8 ± 2.0 *
Serum IL-6 (pg/mL)	0.68 ± 0.1	2.1 ± 0.3	3.7 ± 2.2 *
Serum CRP (ng/mL)	1.2 ± 0.7	1.6 ± 0.5	6.1 ± 0.8 ***†
Serum Ammonia	11.5 ± 0.09	11.62 ± 0.1	11.73 ± 0.04 *

BF, body fat, BM, body mass, BMI, body mass index, CRP, c-reactive protein, FFM, fat free mass, HGS, hand grip strength, IL-6, interleukin-6, WBFM, whole body fat mass. * = *p* < 0.05 significantly different from CON. *** = *p* < 0.001 significantly different from CON. † = *p* < 0.001 significantly different from NAFLD.

## Data Availability

Data are contained within this article.

## Supplementary Materials

The following supporting information can be downloaded at: https://www.mdpi.com/article/10.3390/cells11071098/s1, Figure S1: Representative images of myotubes taken in response to 24-h treatments with serum from CON, NAFLD and ESLD participants.
