# Supplementary material for: The Effect of Ex Vivo Human Serum from Liver Disease Patients on Cellular Protein Synthesis and Growth
Source: Cells. 2022 Mar 24;11(7):1098. doi: 10.3390/cells11071098 (PMC8997893; doi:10.3390/cells11071098)
Supplement: Supplementary file 1 [file cells-11-01098-s001.zip › cells-1631079-supplementary.pdf]

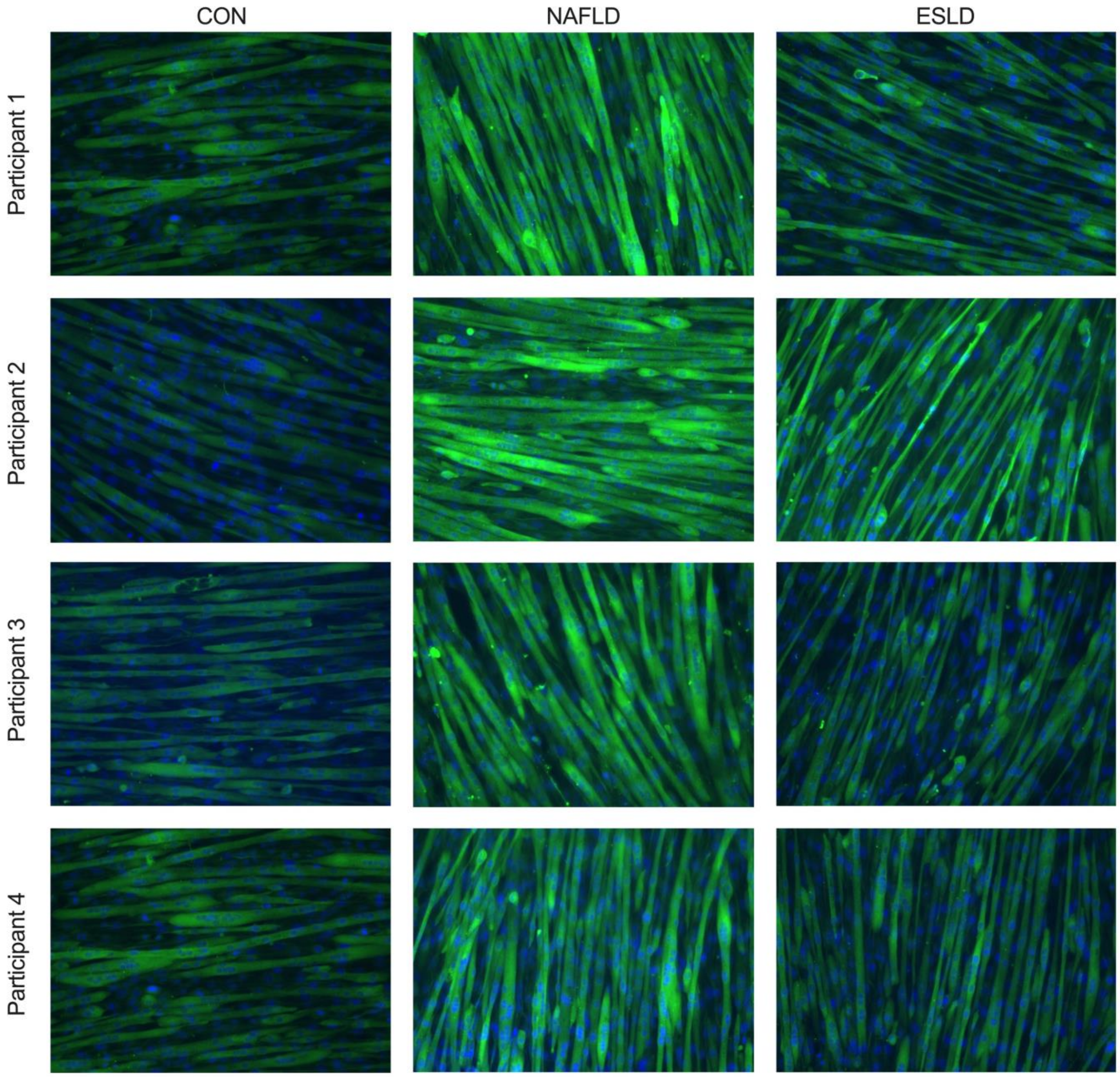

Supplementary Figure S1. Representative images of myotubes taken in response to 24-h treatments with serum from CON, NAFLD and ESLD participants. Images represent the merged images of DAPI (blue) and desmin (green) channels. Images represent myotubes in response to treatment with serum from each individual participant at one technical replicate.
